# Supplementary material for: Fetal and trophoblast PI3K p110α have distinct roles in regulating resource supply to the growing fetus in mice
Source: eLife. 2019 Jun 26;8:e45282. doi: 10.7554/eLife.45282 (PMC6634971; doi:10.7554/eLife.45282)
Supplement: Figure 1—source data 1. — * Note for Cyp19Cre mutants (Het-P and Hom-P) the frequency is actually ~half than stated, due to mosaic activity of this Cre line. [file elife-45282-fig1-data1.docx]

**Figure 1-source data 1. Summary of the mouse strains and experimental crosses used in the study.** * Note for *Cyp19*Cre mutants (Het-P and Hom-P) the frequency is actually ~half than stated, due to mosaic activity of this Cre line.

| Paternal  genotype | Maternal  genotype | Conceptus genotypes  (for experiments) | Frequency* | Used for |
| --- | --- | --- | --- | --- |
| *Meox2*Cre (het) | *Pik3ca*^Fl/Fl^ | *Pik3ca*^Fl/+^ = WT  *Meox2*Cre; *Pik3ca*^Fl/+^ = Het-F | 1:2 | Day 19 collection for fetal weight and placental transport phenotype |
| *Pik3ca* null (het); *Meox2*Cre (het) | *Pik3ca*^Fl/Fl^ | *Pik3ca*^Fl/+^ = WT  *Meox2*Cre; *Pik3ca*^Fl/+^ = Het-F  *Pik3ca* null het and *Pik3ca*^Fl/+^ = Het-U  *Meox2*Cre; *Pik3ca*^Fl/+^ and *Pik3ca* null het = Hom-F | 1:4 | D10-13 collection for conceptus viability |
| *Pik3ca*^Fl/Fl^ | *Pik3ca* null (het); *Cyp19*Cre (het) | *Pik3ca*^Fl/+^ = WT  *Cyp19*Cre; *Pik3ca*^Fl/+^ = Het-P  *Pik3ca* null het and *Pik3ca*^Fl/+^ = Het-U  *Cyp19*Cre; *Pik3ca*^Fl/+^ and *Pik3ca* null het = Hom-P | 1:4 | Day 19 collection for conceptus viability, fetal weight and placental transport phenotype |
