# Supplementary material for: Fetal and trophoblast PI3K p110α have distinct roles in regulating resource supply to the growing fetus in mice
Source: eLife. 2019 Jun 26;8:e45282. doi: 10.7554/eLife.45282 (PMC6634971; doi:10.7554/eLife.45282)
Supplement: Table 1—source data 1. — Frequency of viable fetuses in a litter are displayed, with data from n = 15 litters. Offspring genotypes were determined by conventional PCR, and in the case of Cyp19Cre mutants, additionally by qRT-PCR to identify those with a sufficient level of Pik3ca deletion (frequency is in parentheses). When the cut off for Pik3ca deletion in the placenta using qRT-PCR was applied (<65% for Het-P and <30% for Hom-P), the frequency of Cyp19Cre mutants was ~50% less. [file elife-45282-table1-data1.docx]

**Table 1-source data 1. Deleting the remaining p110α from the trophoblast in Hom-P does not affect fetal viability at day 19 of pregnancy.** Frequency of viable fetuses in a litter are displayed, with data from n=15 litters. Offspring genotypes were determined by conventional PCR, and in the case of *Cyp19*Cre mutants, additionally by qRT-PCR to identify those with a sufficient level of *Pik3ca* deletion (frequency is in parentheses). When the cut off for *Pik3ca* deletion in the placenta using qRT-PCR was applied (<65% for Het-P and <30% for Hom-P), the frequency of *Cyp19*Cre mutants was ~50% less.

|  | WT | Het-P | Het-U | Hom-P |
| --- | --- | --- | --- | --- |
| Frequency by DNA genotyping (by qRT-PCR) | 26% | 23% (14%) | 19% | 32% (16%) |
