# Supplementary material for: Fetal and trophoblast PI3K p110α have distinct roles in regulating resource supply to the growing fetus in mice
Source: eLife. 2019 Jun 26;8:e45282. doi: 10.7554/eLife.45282 (PMC6634971; doi:10.7554/eLife.45282)
Supplement: Table 2—source data 1. — Hom-P * versus WT or † versus Het-P. *p<0.05 and ***p<0.001, †p<0.05 and †††p<0.001, unpaired t test. Conceptus weights are from n ≥ 15, Lz and Jz volume from n ≥ 6 and Lz morphology from n ≥ 4 per genotype on day 19 of pregnancy. Data are presented as means ± SEM. [file elife-45282-table2-data1.docx]

**Table 2-source data 1. The effect of deleting the remaining p110α from the trophoblast in Hom-P on feto-placental growth relative to WT and Het-P.** Hom-P * *versus* WT or † *versus* Het-P. *P < 0.05 and ***P < 0.001, †P < 0.05 and †††P < 0.001, unpaired t test. Conceptus weights are from n≥15, Lz and Jz volume from n≥6 and Lz morphology from n≥4 per genotype on day 19 of pregnancy. Data are presented as means ± SEM.

|  | WT | Het-P | Hom-P |
| --- | --- | --- | --- |
| Fetus (mg) | 1155±20 | 1152±25 | 994±22***††† |
| Placenta (mg) | 93±3 | 101±5 | 89±3† |
| Labyrinthine zone (mm^3^) | 47±2 | 46±4 | 36±2*** |
| Junctional zone (mm^3^) | 33±5 | 34±9 | 30±2 |
| Maternal blood spaces (mm^3^) | 14±2 | 9±1 | 11±1† |
| Trophoblast (mm^3^) | 28±2 | 34±2 | 22±2††† |
| Fetal capillaries (mm^3^) | 5±1 | 3±1 | 4±1*† |
| Barrier thickness (μm) | 3.5±0.1 | 3.7±0.1 | 3.9±0.1* |
| Surface area (mm^2^) | 15±1 | 9±1 | 10±1*** |
