# Supplementary material for: Fetal and trophoblast PI3K p110α have distinct roles in regulating resource supply to the growing fetus in mice
Source: eLife. 2019 Jun 26;8:e45282. doi: 10.7554/eLife.45282 (PMC6634971; doi:10.7554/eLife.45282)
Supplement: Figure 5—source data 1. — Placental transport of 3H-methyl-D glucose (MeG) and 14C-amino isobutyric acid (MeAIB) relative to surface area available or to fetal weight on day 19 of pregnancy is shown as a ratio of WT values. Hom-P * versus WT or † versus Het-P. *p<0.05, †p<0.05 and †††p<0.001, unpaired t test. DPM = disintegrations per minute. Data are from n ≥ 15 and presented as means ± SEM. [file elife-45282-fig5-data1.docx]

**Figure 5-source data 1. The effect of deleting the remaining p110α from trophoblast in Hom-P on placental transport capacity relative to WT and Het-P.** Placental transport of ^3^H-methyl-D glucose (MeG) and ^14^C-amino isobutyric acid (MeAIB) relative to surface area available or to fetal weight on day 19 of pregnancy is shown as a ratio of WT values. Hom-P * *versus* WT or † *versus* Het-P. *P < 0.05, †P < 0.05 and †††P < 0.001, unpaired t test. DPM = disintegrations per minute. Data are from n≥15 and presented as means ± SEM.

|  | WT | Het-P | Hom-P |
| --- | --- | --- | --- |
| MeG DPM /mm^2^ SA (ratio to WT) | 1.0±0.1 | 1.8±0.1 | 1.4±0.1*† |
| MeAIB DPM /mm^2^ SA (ratio to WT) | 1.0±0.1 | 2.1±0.2 | 1.2±0.1††† |
| MeG DPM/g fetus (ratio to WT) | 1.0±0.1 | 1.1±0.1 | 1.1±0.1 |
| MeG DPM/g fetus (ratio to WT) | 1.0±0.1 | 1.2±0.1 | 0.9±0.1 |
