# Supplementary material for: Fetal and trophoblast PI3K p110α have distinct roles in regulating resource supply to the growing fetus in mice
Source: eLife. 2019 Jun 26;8:e45282. doi: 10.7554/eLife.45282 (PMC6634971; doi:10.7554/eLife.45282)
Supplement: Figure 7—source data 2. [file elife-45282-fig7-data2.docx]

**Figure 7-source data 2. Primer sequence for RT-qPCR, PCR screening and CRISPR gRNAs target**

| **RT-qPCR primers** | | | |
| --- | --- | --- | --- |
| **Gene** | **Sequence** | | |
| *Aldh1a3* | **F -** ACTGGAGCTAGGAGGCAAGAAC | | |
|  | **R -** GTAGACCTGCTCTTCCACGAAC | | |
| *Sult1e1* | **F -** CTTCCAGGAGATGAAGAACAATCC | | |
|  | **R -** GGAAGTGGTTCTTCCAGTCTCC | | |
| *Prl5a1* | **F -** ACACTTCATCCCGCTCTGTC | | |
|  | **R -** CAGAAGGTTTTTCCAGGCAGC | | |
| *Mgst1* | **F -** GGTGAAAAGTCCCAGAAGTGC | | |
|  | **R -** GCCATCAACACCTCATTGTCC | | |
| *Cdx2* | **F -** GCTCTTTGCCAGGACTGACT | | |
|  | **R -** CAGCCACCTTGGCTCAAGTA | | |
| *Pdlim3* | **F -** TCATGCTGATGCACAGGACA | | |
|  | **R -** GCGGTACCAATGGGTTTGAA | | |
| *Creb3l1* | **F -** GTCCCCAGACACTTGGACTG | | |
|  | **R -** GAACAGTGGTTGCGTTGGTC | | |
| *Cited2* | **F -** ATCGGCTGTCCCTCTATGTG | | |
|  | **R -** CCATTTCCAGTCCTTCCGTCT | | |
| *Selenbp1* | **F -** GTGAGCAGTTTGCACACCAG | | |
|  | **R -** GGCTTCTCCCATGTCCCTTT | | |
| *Nov* | **F -** AACAGGAATCGCCAGTGTGA | | |
|  | **R -** TTTCTTGGTGCGGAGACACT | | |
| *B2m* | **F -** TTCTGGTGCTTGTCTCACTGA | | |
|  | **R -** CAGTATGTTCGGCTTCCCATTC | | |
| *Gzmc* | **F -** TGTGAGTCCCAGTTCCAAAGTT | | |
|  | **R -** TGCTCTTTTACACACAAGCGG | | |
| *Gzmd* | **F -** TGTCTGTGGATATTAAGGGGAACA | | |
|  | **R -** TGTCATTGAGCTTTGGACAGAG | | |
| *Gzme* | **F -** TGTCTCCTTTGCTCTCCTTCAA | | |
|  | **R -** ATCCACAGACTTAACAAACGCC | | |
| *Gzmf* | **F -** ACATCCATCAATGCAACACAAAG | | |
|  | **R -** TTGTTACACACGAGGGGTCC | | |
| *Gzmg* | **F -** TTTTAATAGAAAGCATGGCACCA | | |
|  | **R -** GGCCTCACAGCTTTAGTTCTC | | |
| *Mpzl1* | **F -** GGCTCCACGTGGTGGAAATA | | |
|  | **R -** TGACTGGGCCCCGGTATAAT | | |
| *Mpzl2* | **F -** TCTCCGTGGGTAAACTGTGC | | |
|  | **R -** TAGGACAAAGGGCTGTGAGC | | |
| *Tmsb10* | **F -** CGGAAGGCGCGGGAT | | |
|  | **R -** TCAATGGTCTCTTTGGTCGGC | | |
| *Aqp5* | **F -** ATCCATTGGCTTGTCGGTCA | | |
|  | **R -** GTCCTACCCAGAAGACCCAGT | | |
| *Gapdh* | **F -** GGGAAATGAGAGAGGCCCAG | | |
|  | **R -** GAACAGGGAGGAGCAGAGAG | | |
| *Sdha* | **F -** TGGTGAGAACAAGAAGGCATCA | | |
|  | **R -** CGCCTACAACCACAGCATCA | | |
| *Pik3ca* ex17-18 | **F -** GCTGCAGTTCAACAGCCACA | | |
|  | **R -** GCAGGACCGAGTGAACAGGT | | |
| *Pik3ca* ex19-20 | **F -** AGGGAGCACAAGAGTACACCA | | |
|  | **R -** GGCATGCTGCCGAATTGCTA | | |
| **PCR screening primers** | | |  |
| Pik3ca | **F -** ATCCCATGGAGAAAGAGACCAT | | |
|  | **R -** GGTTACTTCTTTTGTAGGTTGCAC | | |
| **CRISPR gRNAs target sequences** | |  | |
| *Pik3ca* (exon 18-19) | Upstream exon 18 | CACCGCGGAGTGGCTGCGGCCAAAC | |
|  | Upstream exon 18 | CACCGTCAGCCGTATTTCCCCAGTT | |
|  | Downstream exon 19 | CACCGTCTCTTCTGTCCGATGTCG | |
|  | Downstream exon 19 | CACCGTGGTCACGCACACACGGTCT | |
