# Supplementary material for: Fetal and trophoblast PI3K p110α have distinct roles in regulating resource supply to the growing fetus in mice
Source: eLife. 2019 Jun 26;8:e45282. doi: 10.7554/eLife.45282 (PMC6634971; doi:10.7554/eLife.45282)
Supplement: Transparent reporting form [file elife-45282-transrepform.docx]

***eLife’s* transparent reporting form**

We encourage authors to provide detailed information *within their submission* to facilitate the interpretation and replication of experiments. Authors can upload supporting documentation to indicate the use of appropriate reporting guidelines for health-related research (see [EQUATOR Network](http://www.equator-network.org/%20)), life science research (see the [BioSharing Information Resource](https://biosharing.org/)), or the [ARRIVE guidelines](http://www.plosbiology.org/article/info:doi/10.1371/journal.pbio.1000412) for reporting work involving animal research. Where applicable, authors should refer to any relevant reporting standards documents in this form.

If you have any questions, please consult our Journal Policies and/or contact us: [editorial@elifesciences.org](mailto:editorial@elifesciences.org).

**Sample-size estimation**

- You should state whether an appropriate sample size was computed when the study was being designed
- You should state the statistical method of sample size computation and any required assumptions
- If no explicit power analysis was used, you should describe how you decided what sample (replicate) size (number) to use

Please outline where this information can be found within the submission (e.g., sections or figure legends), or explain why this information doesn’t apply to your submission:

No explicit power analysis was used to predetermine sample size. However, the number of conceptuses for growth and placental transport data were informed by our previous studies showing 8 litters or more are typically required to detect a difference in means between groups. From those, > 4 samples per genotype were used in morphological and molecular analyses (RNA-seq and qPCR), which is standard in the field. For the CRISPR/CAS9 experiments, > 4 samples per cell type and genotype were used. We state that no explicit power analyses were used to predetermine sample size, in the statistical section of the paper. The information about the number of samples per group is detailed in the legends of figures and footnotes of tables.

**Replicates**

- You should report how often each experiment was performed
- You should include a definition of biological versus technical replication
- The data obtained should be provided and sufficient information should be provided to indicate the number of independent biological and/or technical replicates
- If you encountered any outliers, you should describe how these were handled
- Criteria for exclusion/inclusion of data should be clearly stated
- High-throughput sequence data should be uploaded before submission, with a private link for reviewers provided (these are available from both GEO and ArrayExpress)

Please outline where this information can be found within the submission (e.g., sections or figure legends), or explain why this information doesn’t apply to your submission:

The majority of data presented are from in vivo procedures where the number of replicates indicates the number of conceptuses per genotype. For the cell culture work using CRISPR/CAS9 experiments, the number of replicates also indicates the number of clones per genotype. This information can be found in each figure legend.

However, in the case of gene expression analyses by qRT-PCR, experiments were performed in duplicate and sample means were used in statistical analyses. This is stated in the ‘Gene expression by qRT-PCR’ section of the materials and methods.

Details of sample sizes and biological and technical replication are in figure legends and table footnotes.

Due to the mosaic activity of the *Cyp19*Cre (Wenzel and Leone, 2007) a cut-off for *Pik3ca* deletion in the placenta using qRT-PCR was set for <65% Het-P and <30% for Hom-P. This is stated in the materials and methods section entitled, ‘mice and genotyping’.

Our RNAseq data are included as supplementary Table 5 and are accessible on this link <https://www.ncbi.nlm.nih.gov/geo/query/acc.cgi?acc=GSE126046> with the token/password: ylmtqsckpzmnhyd.

**Statistical reporting**

- Statistical analysis methods should be described and justified
- Raw data should be presented in figures whenever informative to do so (typically when N per group is less than 10)
- For each experiment, you should identify the statistical tests used, exact values of N, definitions of center, methods of multiple test correction, and dispersion and precision measures (e.g., mean, median, SD, SEM, confidence intervals; and, for the major substantive results, a measure of effect size (e.g., Pearson's r, Cohen's d)
- Report exact p-values wherever possible alongside the summary statistics and 95% confidence intervals. These should be reported for all key questions and not only when the p-value is less than 0.05.

Please outline where this information can be found within the submission (e.g., sections or figure legends), or explain why this information doesn’t apply to your submission:

Statistical analyses are described in the materials and methods. In particular, data were considered normally distributed and analysed using unpaired and paired t tests with Excel, as required or two-way ANOVA with GraphPad Prism 7 (for CRISPR/CAS9 experiments: cell type, genotype). Data are presented as mean ± SEM and numbers, statistical tests and p values are detailed in figure legends and table footnotes.

(For large datasets, or papers with a very large number of statistical tests, you may upload a single table file with tests, Ns, etc., with reference to sections in the manuscript.)

**Group allocation**

- Indicate how samples were allocated into experimental groups (in the case of clinical studies, please specify allocation to treatment method); if randomization was used, please also state if restricted randomization was applied
- Indicate if masking was used during group allocation, data collection and/or data analysis

Please outline where this information can be found within the submission (e.g., sections or figure legends), or explain why this information doesn’t apply to your submission:

Randomization was not used in our studies. Analyses of placental morphology and in situ cell death staining were conducted blinded to the genotype. These information are included in the statistics section of the materials and methods.

**Additional data files (“source data”)**

- We encourage you to upload relevant additional data files, such as numerical data that are represented as a graph in a figure, or as a summary table
- Where provided, these should be in the most useful format, and they can be uploaded as “Source data” files linked to a main figure or table
- Include model definition files including the full list of parameters used
- Include code used for data analysis (e.g., R, MatLab)
- Avoid stating that data files are “available upon request”

Please indicate the figures or tables for which source data files have been provided:

There are no additional data files for our study.
